# Representation of Women on National Institutes of Health Study Sections

**DOI:** 10.1001/jamanetworkopen.2020.37346

**Published:** 2021-02-15

**Authors:** Anna Volerman, Vineet M. Arora, John F. Cursio, Helen Wei, Valerie G. Press

**Affiliations:** 1 Section of General Internal Medicine, Department of Medicine, The University of Chicago, Chicago, Illinois; 2 Section of Academic Pediatrics, Department of Pediatrics, The University of Chicago, Chicago, Illinois; 3 Department of Public Health Sciences, The University of Chicago, Chicago, Illinois; 4 Pritzker School of Medicine, The University of Chicago, Chicago, Illinois

## Abstract

This cross-sectional study examines the proportion of women represented on National Institutes of Health study sections and whether there is an association with research funding levels.

## Introduction

Disparities in gender representation among researchers are well described throughout the career trajectory, including grant awards.^1,2,3^ This study aimed to evaluate whether differences exist in gender representation on National Institutes of Health (NIH) study sections, which help determine funding.

## Methods

This cross-sectional study examined participants on chartered and special emphasis NIH study sections during one review cycle from May 15 to July 15, 2019. The University of Chicago institutional review board deemed this study exempt because it used publicly available data. This study followed the Strengthening the Reporting of Observational Studies in Epidemiology (STROBE) reporting guideline.

Data were extracted about study section reviewers (name, degree, academic rank, temporary membership, chair), scientific review officers (SROs; name), and institutes, centers, or offices (hereafter referred to as *institutes*; chair name, total funding, number of awards, award amount, and success rate for research grants and projects in 2018).

Individuals’ gender presentation (man or woman) was determined through name-based internet searches for pictures and pronouns. If unconfirmed, gender was assigned using software (Genderize.io [Demografix ApS]; threshold = 60%). χ^2^ tests were used to compare gender distributions between institutes. Univariate and multivariate logistic regressions were applied to determine the likelihood of having women on study sections based on institute and section characteristics. Significance was defined by *P* < .05 using 2-sided tests. Data analyses were performed using SAS version 9.4 (SAS Institute) in August 2020.

## Results

A total of 367 study sections convened with 8817 participants analyzed (Table 1). Of these, 3432 were female (38.9%). Among the 25 institutes, the mean (SD) proportion of women reviewers was 39.0% (15.1%). Women constituted more than half of reviewers in 4 institutes (16%). Less than half of study sections had a woman SRO (49.0% [180 of 367]) or chair (37.3% [137 of 367]). Nine institutes (36%) were chaired by a woman.

**Table 1.** Gender Representation of Participants on National Institutes of Health Study Sections by Institutes, Center, and Office, May 15 to July 15, 2019^a^

Institute, center, or office	Study sections that met in 1 review cycle, No.	Participants on all study sections, total No.^b^	Participants per study section, mean (SD), No.	Proportion of women participants on study sections, mean (SD) [range], %	No. (%)			*P* value to compare gender representation on study sections
					Woman as scientific review officer for study sections	Woman as chair of study sections	Women participants on study sections	
Total	367	8817	24.0 (13.4)	39.0 (15.1) [0.0-100.0]	180 (49.0)	137 (37.3)	3432 (38.9)	<.001
CSR^c^	170	4907	28.9 (7.1)	38.6 (12.3) [14.3-79.4]	83 (48.8)	54 (31.8)	1906 (38.8)	<.001
NCATS	1	22	22.0 (NA)	22.7 (0.0) [22.7-22.7]	0	0	5 (22.7)	.01
NCCIH^c^	5	158	31.6 (9.9)	40.5 (7.6) [33.3-48.8]	7 (100)^d^	0	66 (41.8)	.51
NCI	28	675	24.1 (8.8)	38.3 (18.6) [10.5-93.3]	7 (25.0)	14 (50.0)	255 (37.8)	<.001
NEI^c^	2	40	20.0 (9.9)	34.0 (6.2) [29.6-38.5]	0	0	13 (32.5)	.58
NIA	7	122	17.4 (9.3)	44.9 (15.0) [16.7-60.0]	5 (71.4)	2 (28.6)	61 (50.0)	.61
NIAAA	8	86	10.8 (6.0)	45.7 (10.7) [33.3-63.2]	4 (50.0)	5 (62.5)	43 (50.0)	.85
NIAID	10	178	17.8 (6.1)	33.5 (11.3) [18.5-57.1]	3 (30.0)	3 (30.0)	58 (32.6)	.44
NIAMS	3	69	23.0 (4.4)	30.4 (12.2) [16.7-40.0]	1 (33.3)	3 (100)	22 (31.9)	.25
NIBIB	4	60	15.0 (13.3)	16.1 (21.1) [0.0-44.4]	2 (50.0)	1 (25.0)	11 (18.3)	.05
NICHD^c^	7	97	13.9 (5.2)	56.9 (21.7) [20.0-83.3]	6 (85.7)	2 (28.6)	57 (58.8)	.02
NIDCD^c^	2	48	24.0 (19.8)	52.6 (3.7) [50.0-55.3]	1 (50.0)	0	26 (54.2)	.77
NIDCR^c^	8	120	15.0 (10.0)	37.7 (16.0) [16.7-66.7]	6 (75.0)	6 (75.0)	47 (39.2)	.24
NIDA^c^	17	208	12.2 (11.3)	34.9 (15.0) [15.8-66.7]	6 (35.3)	9 (52.9)	72 (34.6)	.67
NIDDK	10	237	23.7 (9.7)	40.7 (14.2) [21.9-60.0]	5 (50.0)	2 (20.0)	90 (38.0)	.10
NIEHS^c^	2	56	28.0 (15.6)	39.1 (13.7) [29.4-48.7]	1 (50.0)	1 (50.0)	24 (42.9)	.18
NHGRI	5	56	11.2 (3.4)	45.5 (16.7) [30.0-63.6]	0	2 (40.0)	25 (44.6)	.35
NHLBI	20	341	17.1 (10.8)	34.4 (10.9) [16.7-50.0]	10 (50.0)	7 (35.0)	218 (37.5)	.81
NIGMS	14	455	32.5 (46.1)	39.4 (16.7) [18.8-66.7]	7 (50.0)	4 (28.6)	156 (34.3)	.001
NIMH	16	305	19.1 (8.5)	40.2 (21.3) [0.0-75.0]	10 (62.5)	6 (37.5)	136 (44.6)	<.001
NIMHD	1	21	21.0 (NA)	61.9 (0.0) [61.9-61.9]	0	1 (100)	13 (61.9)	.28
NINDS	18	432	24.0 (11.8)	34.4 (13.5) [14.3-64.7]	11 (61.1)	10 (55.6)	150 (34.7)	.04
NINR	4	38	9.5 (5.1)	75.8 (25.4) [40.0-100.0]	1 (25.0)	3 (75.0)	30 (79.0)	.10
NLM^c^	4	52	13.0 (6.9)	43.5 (13.3) [25.0-55.6]	4 (100)	1 (25.0)	23 (44.2)	.61
Office of director	1	34	34.0 (NA)	44.1 (0.0) [44.1-44.1]	0	1 (100.0)	15 (44.1)	.49

Abbreviations: CSR, Center for Scientific Review; NCATS, National Center for Advancing Translational Sciences; NCCIH, National Center for Complementary and Integrative Health; NCI, National Cancer Institute; NEI, National Eye Institute; NHGRI, National Human Genome Research Institute; NHLBI, National Heart, Lung, and Blood Institute; NIA, National Institute on Aging; NIAAA, National Institute on Alcohol Abuse and Alcoholism; NIAID, National Institute of Allergy and Infectious Diseases; NIAMS, National Institute of Arthritis and Musculoskeletal and Skin Diseases; NIBIB, National Institute of Biomedical Imaging and Bioengineering; NICHD, National Institute of Child Health and Human Development; NIDA, National Institute on Drug Abuse; NIDCD, National Institute on Deafness and Other Communication Disorders; NIDCR, National Institute of Dental and Craniofacial Research; NIDDK, National Institute of Diabetes and Digestive and Kidney Diseases; NIEHS, National Institute of Environmental Health Sciences; NIGMS, National Institute of General Medical Sciences; NIMH, National Institutes of Mental Health; NIMHD, National Institute of Minority Health and Health Disparities; NINDS, National Institute of Neurological Disorders and Stroke; NINR, National Institute of Nursing Research; NLM, National Library of Medicine.

^a^ During one grant review cycle, 367 chartered and special emphasis study sections were convened; integrated review groups and study sections for business and fellowship grants were excluded from this analysis. There were a total of 8820 total participants (unique = 8346). Gender was identified using name-based internet searches to find pictures and/or pronouns. For 23 participants, gender could not be confirmed. Using software (Genderize.io, probability threshold = 60%), gender was assigned for 20 of these participants. Gender could not be determined for 3 participants (unique = 2). Thus, the analysis included 8817 participants. Data about study section participants was obtained from publicly available meeting rosters at the NIH Scientific Review Group Roster Index. Data about NIH institutes, centers, and offices was obtained from the National Institutes of Health Data Book.

^b^ This column represents total participants on the study sections (not unique participants).

^c^ The chair of this institute, center, or office was a woman.

^d^ In this institute, 2 different study sections had 2 scientific review officers.

Overall, reviewers were more likely to be men than women (61.1% vs 38.9%, *P* < .001). Six institutes (24%) were more likely to have men as reviewers and one was more likely to have women. Across all institutes (Table 2), women reviewers were more likely to hold lower academic rank (instructor or none: OR, 1.39; 95% CI, 1.19-1.61; *P* < .001; assistant professor: OR, 1.86; 95% CI, 1.60-2.15; *P* < .001; associate professor: OR, 1.63; 95% CI, 1.47-1.80; *P* < .001) and nondoctoral degrees (OR, 1.61; 95% CI, 1.45-1.78; *P* < .001). Women were more likely to be temporary members (OR, 1.02; 95% CI, 1.01-1.03; *P* < .001) and less likely to be on standing study sections (OR, 0.996; 95% CI, 0.995-0.998; *P* < .001).

**Table 2.** Characteristics Associated With Gender Representation on National Institutes of Health Study Sections^a^

Characteristic	Univariate		Multivariate	
	OR (95% CI)	*P* value	OR (95% CI)	*P* value
Academic rank				
Instructor or none	1.39 (1.19-1.61)	<.001	1.43 (1.17-1.75)	.001
Assistant professor	1.86 (1.60-2.15)	<.001	1.81 (1.45-2.26)	<.001
Associate professor	1.63 (1.47-1.80)	<.001	1.49 (1.27-1.75)	<.001
No doctoral degree (MD or PhD)	1.61 (1.45-1.78)	<.001	1.72 (1.47-2.02)	<.001
Temporary member on study section	1.02 (1.01-1.03)	<.001	0.99 (0.77-1.26)	.91
Standing study section	0.996 (0.995-0.998)	<.001	0.76 (0.63-0.92)	.006
Woman as study section chair	0.98 (0.97-0.999)	.03	1.40 (1.20-1.63)	<.001
Woman as scientific review officer	0.98 (0.96-1.005)	.13	1.02 (0.88-1.19)	.75
Woman as institute, center, or office chair	1.17 (1.00-1.38)	.049	0.98 (0.59-1.62)	.93
Total funding of institute, center, or office	0.92 (0.87-0.97)	.002	1.02 (0.26-4.02)	.98
No. of research grants awarded	0.94 (0.91-0.97)	<.001	0.89 (0.43-1.85)	.76
Research grant average funding	0.99 (0.96-1.03)	.61	0.98 (0.88-1.08)	.64
Research project				
Award amount	0.75 (0.56-1.02)	.06	1.61 (0.23-11.09)	.63
Success rate	0.995 (0.98-1.01)	.33	0.99 (0.96-1.03)	.75

Abbreviations: NIH, National Institutes of Health; OR, odds ratio.

^a^ Univariate and multivariate logistic regression models were applied to determine the likelihood of having a woman on a study section based on NIH institute and study section characteristics (eg, academic rank, degree, temporary membership, standing study section, leadership, grants, funding).

Study sections chaired by women were less likely to have women as reviewers (OR, 0.98; 95% CI, 0.97-0.999; *P* = .03), whereas study sections within an institute with a woman chair were more likely to have women as reviewers (OR, 1.17; 95% CI, 1.00-1.38; *P* = .049). There were lower odds of having women as reviewers among institutes with higher total funding (OR = 0.92, 95% CI = 0.87-0.97, *P* = .002) and higher number of research grant awards (OR = 0.94, 95% CI = 0.91-0.97, *P* < .001). Institutes’ research grant average funding, project award amount, and project success rate were not associated with the proportion of women reviewers on study sections.

## Discussion

To our knowledge, this study is the first to describe differences in gender representation among NIH study sections. Men were more likely to be reviewers and chairs. Women were more likely to have temporary affiliations and serve on study sections with lower total funding and research grants awarded, suggesting less influential opportunities to impact the nation’s research agenda. These findings may partially explain gender bias in peer review and differences in funding and promotion^4,5,6^ with potential ramifications for gender representation in academia.

Intentional efforts to increase gender representation on study sections are critical. NIH’s Early Career Reviewer Program has helped diversify the reviewer pool. Creating study sections through committees or applications, rather than individual recruiters, may further reduce disparities. Implicit bias training may be important for individuals selecting reviewers. At minimum, data about study section composition should be routinely reviewed and shared.

This study had some limitations. Because only one grant cycle was examined, generalizability may be limited. Selection bias could occur since attendance and program officer information were unavailable on meeting rosters. Effect sizes varied and practical impacts of small effects are unknown. Future studies should longitudinally examine gender representation on study sections and evaluate interventions. Deliberate efforts are necessary to reach equitable gender representation on NIH study sections given potential long-term effects on the nation’s research agenda and workforce.

## References

[1] Oliveira DFM, Ma Y, Woodruff TK, Uzzi B Comparison of National Institutes of Health grant amounts to first-time male and female principal investigators. JAMA. 2019;321(9):898-900. doi:10.1001/jama.2018.21944 30835300 PMC6439593

[2] Sege R, Nykiel-Bub L, Selk S Sex differences in institutional support for junior biomedical researchers. JAMA. 2015;314(11):1175-1177. doi:10.1001/jama.2015.8517 26372589

[3] Jagsi R, DeCastro R, Griffith KA, Similarities and differences in the career trajectories of male and female career development award recipients. Acad Med. 2011;86(11):1415-1421. doi:10.1097/ACM.0b013e3182305aa6 21952061

[4] Pohlhaus JR, Jiang H, Wagner RM, Schaffer WT, Pinn VW Sex differences in application, success, and funding rates for NIH extramural programs. Acad Med. 2011;86(6):759-767. doi:10.1097/ACM.0b013e31821836ff 21512358 PMC3379556

[5] Kaatz A, Lee Y-G, Potvien A, Analysis of National Institutes of Health R01 application critiques, impact, and criteria scores: does the sex of the principal investigator make a difference? Acad Med. 2016;91(8):1080-1088. doi:10.1097/ACM.0000000000001272 27276003 PMC4965296

[6] Magua W, Zhu X, Bhattacharya A, Are female applicants disadvantaged in National Institutes of Health peer review? combining algorithmic text mining and qualitative methods to detect evaluative differences in R01 reviewers’ critiques. J Womens Health (Larchmt). 2017;26(5):560-570. doi:10.1089/jwh.2016.6021 28281870 PMC5446598

